# Supplementary figures and images for: Changes of faecal bacterial communities and microbial fibrolytic activity in horses aged from 6 to 30 years old
Source: PLoS One. 2024 Jun 3;19(6):e0303029. doi: 10.1371/journal.pone.0303029 (PMC11146703; doi:10.1371/journal.pone.0303029)

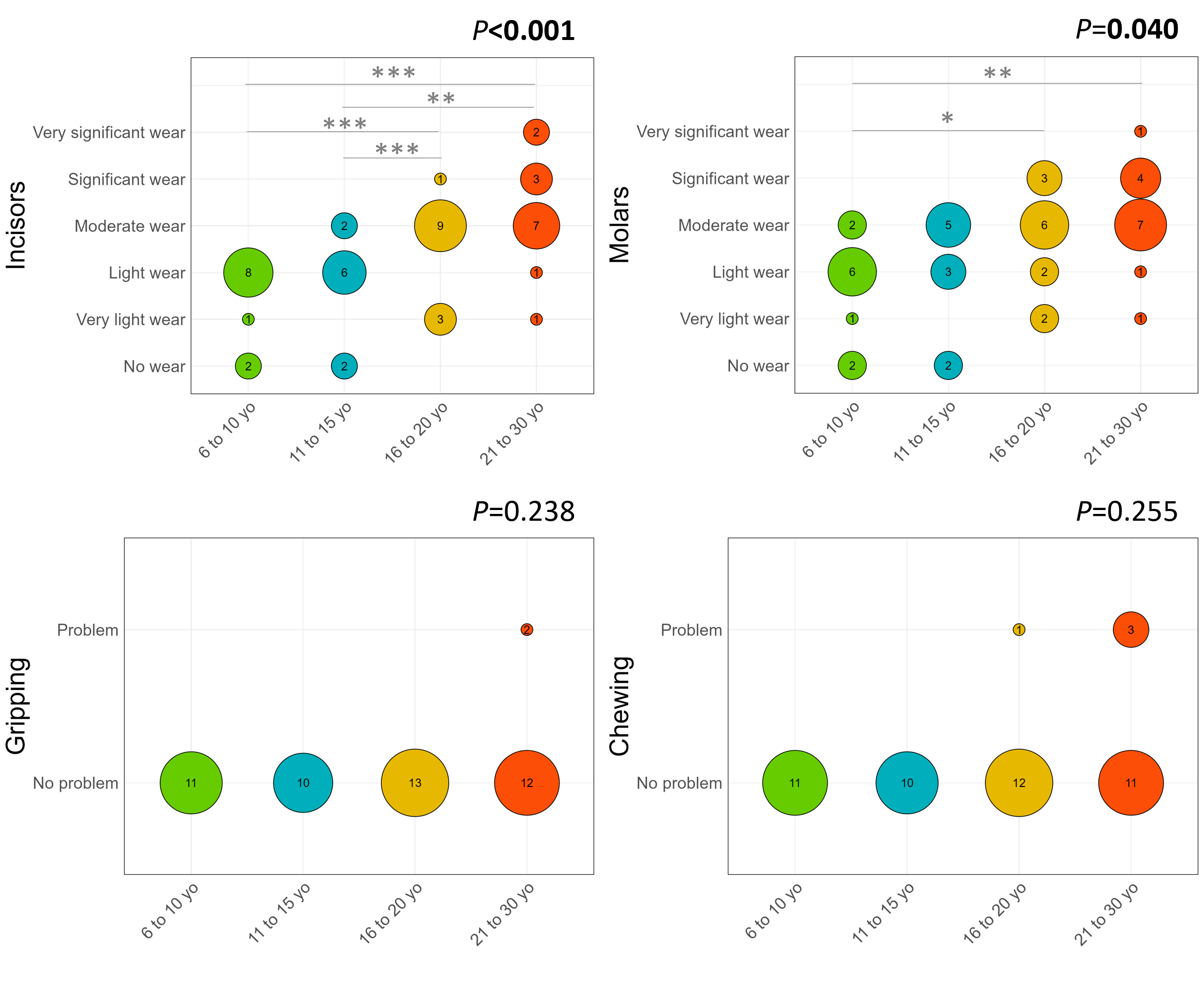

Supplement: S1 Fig — The P values reported correspond to the comparison of each parameter between age categories. Asterisks indicate significant differences between two age categories (*: P < 0.05; **: P < 0.01, ***: P < 0.001). (TIF) [file pone.0303029.s001.tif]

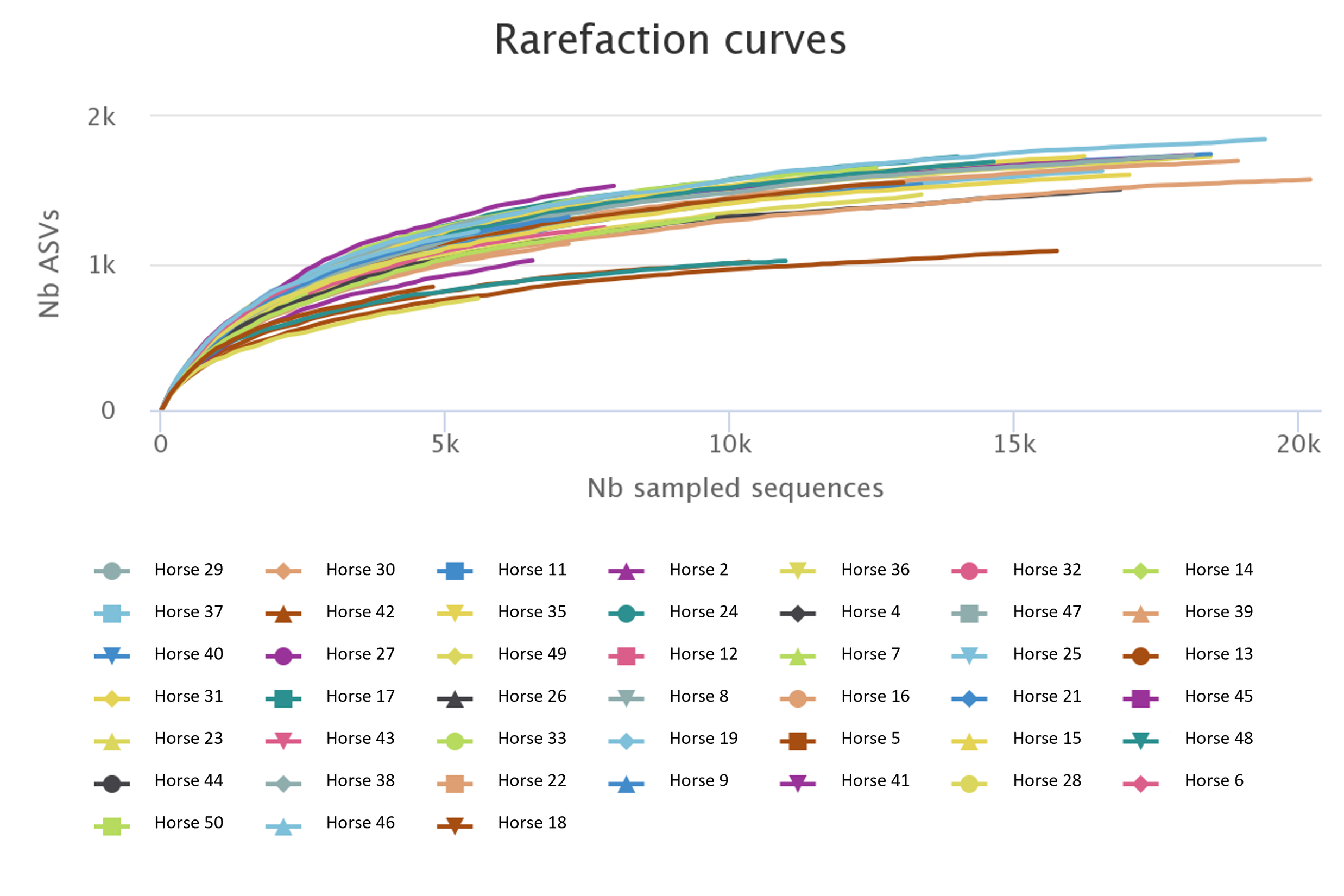

Supplement: S2 Fig — (TIF) [file pone.0303029.s002.tif]
